# Polyphenolic Profile and Varied Bioactivities of Processed Taiwanese Grown Broccoli: A Comparative Study of Edible and Non-Edible Parts

**DOI:** 10.3390/ph13050082

**Published:** 2020-04-28

**Authors:** Thanh Ninh Le, Napat Sakulsataporn, Chiu-Hsia Chiu, Pao-Chuan Hsieh

**Affiliations:** Department of Food Science, National Pingtung University of Science and Technology, Pingtung 91207, Taiwan; ltninh90@gmail.com (T.N.L.); i.ffogg@hotmail.com (N.S.); cschiou@mail.npust.edu.tw (C.-H.C.)

**Keywords:** broccoli, byproducts, antioxidant activity, cytotoxicity, apoptosis, antibacterial activity

## Abstract

Broccoli contains a substantial amount of bioactive compounds such as glucosinolates, phenolics, and essential nutrients, which are positively linked to health-promoting effects. This work aimed to evaluate whether both edible and non-edible parts of broccoli could be effective by examining in vitro antioxidant, cytotoxic, apoptotic, and antibacterial properties of its floret, leaf, and seed extracts (FE, LE, and SE, correspondingly). High-performance liquid chromatography (HPLC) and various assays exhibited strong antioxidant activities of all samples. LE obtained the highest capacity, correlated to its polyphenolic contents. SE exerted significant cytotoxicity against A549, Caco-2, and HepG2 cancer cell lines at low inhibitory concentration (IC)_50_ values (0.134, 0.209, and 0.238 mg/mL, respectively), as tested by 3-(4,5-dimethylthiazol-2-yl)-2,5-diphenyltetrazolium bromide (MTT) assay. Flow cytometry confirmed apoptosis induction of extracts in Caco-2 cells by revealing an increased subG1 population and a decreased mitochondrial membrane potential. The considerable antibacterial efficacy was observed in either LE and SE against *Bacillus subtilis* and *Salmonella typhimurium* (0.39–0.78 mg/mL) using well-agar diffusion and minimum inhibitory concentration (MIC) techniques, along with the weak activity against *Staphylococcus aureus* and *Escherichia coli* (1.56–3.13 mg/mL). The findings suggest that broccoli and its byproducts might serve as a promising source for further development of food or pharmaceutical products.

## 1. Introduction

Over the last decade, the concept of food sustainability has received increased attention mainly owing to the growth of the world’s population and the intensification of pressure on the global food system. Thus, the food system has faced up to a novel challenge: to discover new food ingredients with functional properties of bioactive compounds present in natural matrices [1]. Besides, for the reason that the food processing industry generates an enormous amount of food waste worldwide, food sustainability is also based on the minimization of negative effects of food byproducts on the environment [2]. Currently, there is a growing interest in the recovery of food waste, and upgrading it into high-value byproducts for use as ingredients in new product development [3]. To obtain health-promoting phytochemicals such as vitamins, minerals, antioxidants, or amino acids from food byproducts, several studies have already been carried out on citrus peels, grape seeds, mango peels, pepper stalks, or pomegranate marcs [4,5,6,7]. Furthermore, the main topic of research and innovation funded by the European Union in the Horizon 2020 framework program is developing scientific projects about the valorization of agriculture and food byproducts. The objective of these projects is to achieve extracts, enriched fractions, and isolated compounds, which would be subsequently integrated into food or pharmaceutical product formulations [8].

Plant-derived foods such as vegetables contain high levels of natural antioxidants and bioactive dietary components, so their byproducts could be a valuable source for new product formulations [9]. The genus *Brassica* (Brassicaceae or Cruciferae family), in general, and broccoli, in particular, are rich sources of nitrogen-sulfur derivatives (glucosinolates and isothiocyanates), and they possess high values of phenolics (chlorogenic and sinapic acid derivatives, and flavonols) and essential nutrients (vitamins and minerals) [10,11]. Broccoli (*Brassica oleracea* L. var. *italica*) is one of the most widely consumed vegetables. A diet rich in broccoli has been reported to play an essential role in the prevention of chronic diseases, such as cancer and cardiovascular diseases [12]. However, the main edible part of broccoli is the florets, which make up only 30% of the vegetable’s biomass. This means that around 70% of the plant (mainly leaves) is discarded as non-edible parts. These residues are not valuable commercially, require an additional cost of disposal, and have a detrimental impact on the environment [13]. Moreover, broccoli’s flowering is triggered under the optimal temperatures of 18 to 25 °C. When the temperature exceeds 30 °C, initial floret development is disrupted. Taiwan is located in tropical and subtropical regions, consequently reducing the quality and quantity of broccoli produced during summer [14]. Hence, it might be essential for the food industry to take advantage of broccoli byproducts such as stems, leaves, roots, and seeds as functional product materials, rather than only using the florets.

To date, the use of broccoli byproducts has been applied to flour and fiber [15]. On the other hand, scientific research of chemical composition and biological activities mostly focused on broccoli florets, whereas investigations regarding bioactive potentials of other broccoli parts were generally limited. Several studies demonstrated that broccoli leaves and seeds had good nutritional values, as well as notable antioxidant and antiproliferative capacities [16,17,18]. Nevertheless, to our knowledge, there are no reports available about the mechanism of cancer cell death such as apoptosis induction or cell cycle arrest, and other biologically active properties such as anti-inflammatory or antimicrobial effects of these non-edible parts.

Therefore, the present study was designed to investigate and compare polyphenolic contents and diverse bioactivities of edible and non-edible parts of broccoli on various in vitro models. It is the first to examine the apoptotic and antibacterial activities of broccoli leaves and seeds. Significantly, this study aimed to validate these byproduct extracts as biologically active substances for functional food and nutraceutical applications.

## 2. Results

### 2.1. Antioxidant Activity

At first, broccoli samples were extracted in three different organic solvents (70% methanol, 70% ethanol, and hot water), and the antioxidant potential of the extracts was evaluated to reveal an effective solvent for the further analyses. Broccoli floret, leaf, and seed extracts (FE, LE, and SE, respectively) were investigated using various antioxidant activity assays. As shown in Table 1, the results from the reducing power and ABTS (2,2’-azino-bis-3-ethylbenzothiazoline-6-sulphonic acid) assays indicated that LE achieved the strongest antioxidant capacity in comparison with that of FE and SE, while the highest radical scavenging activity was found in SE by the DPPH (2,2-diphenyl-1-picrylhydrazyl) assay. When comparing the solvents, ethanolic and methanolic extracts exhibited highly active antioxidant capacities, with no notable variations, whereas hot water extracts showed significantly lower values, as presented in Table 1.

Broccoli is known to be rich in antioxidants, mainly phenolic acids. Hence, the determinations of total phenolic content (TPC), total flavonoid content (TFC), and vitamin C content (VCC) were carried out to comparatively confirm antioxidant properties of different broccoli parts. As the values are shown in the Table 2, the maximum TPC and TFC were reported in LE, followed by FE, and least in SE. However, no significant differences were noted between VCC of these extracts. In another aspect, samples extracted by 70% methanol and 70% ethanol had obviously higher TPC, TFC, and VCC than those extracted by hot water, as observed in Table 2.

In addition to the determinations of TPC, TFC, and VVC, the identification of several naturally occurring phenolic components in ethanolic extracts was performed by the high-performance liquid chromatography (HPLC) analysis. Six phenolic and flavonoid compounds, including gallic acid, esculetin, caffeic acid, ferulic acid, myricetin, and quercetin, were characterized. As shown in Table 3, the levels of these compounds were accumulated in FE, LE, and SE with significant variations. Among the compounds, esculetin reached substantial values in all extracts, especially in SE.

After statistically comparing the results of all assays, the highest antioxidant values were indicated by LE independently of the organic solvent used. Samples extracted by 70% methanol and 70% ethanol were more active than those extracted by hot water independently of the broccoli organ used, as well as obtaining higher TPC, TFC, and VVC. Thus, after considering the study objectives and the solvent properties, 70% ethanol was selected to prepare extracts for subsequent experiments.

### 2.2. Cytotoxic and Apoptotic Activities

Three cancer cell lines were used for the cytotoxic activity, along with a normal cell line for the selective activity. As shown in Table 4, the cytotoxicity of FE, LE, and SE exhibited similar results in all cancer cells for 24 h. After 48 h of incubation, these samples showed the stronger cytotoxic efficacy, in which SE obtained the most notable capacity towards A549 (lung carcinoma cells), Caco-2 (colorectal adenocarcinoma cells), and HepG2 (hepatocellular carcinoma cells) with the effective IC_50_ (the 50% growth inhibitory concentration) values of 0.134, 0.209, and 0.238 mg/mL, respectively. In contrast, all extracts were considered to have no effects on FL83B (normal liver cells) growth by the IC_50_ value exceeding the maximum concentration tested. Regarding the positive control, cisplatin, a potent anticancer agent, displayed the lowest IC_50_ values against cancer cells and even normal cells after 48 h of treatment. Among the cancer cells tested, Caco-2 was selected for further analysis based on its stable performance during the experimental period, as well as its functional characteristics.

**Figure 1:**
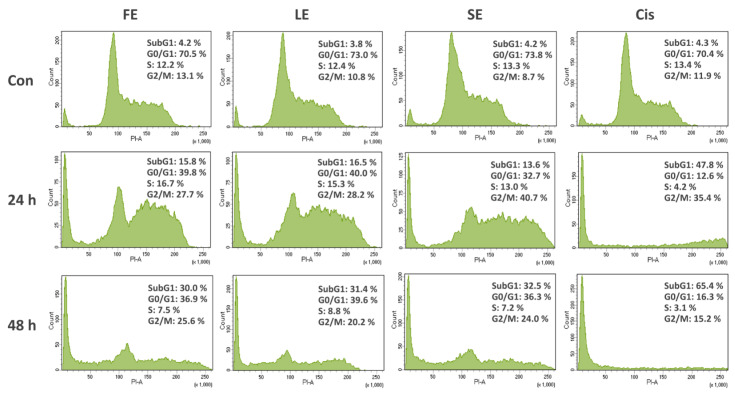
Cell cycle distribution at different phases (SubG1, G0/G1, S, and G2/M) of Caco-2 cells treated with extracts (0.20 mg/mL) and cisplatin (0.02 mg/mL) for 24 and 48 h and analyzed using flow cytometry after staining with PI (propidium iodide). Cis, cisplatin; Con, control (untreated cells). FE, floret extract; LE, leaf extract; SE, seed extract.

**Figure 2:**
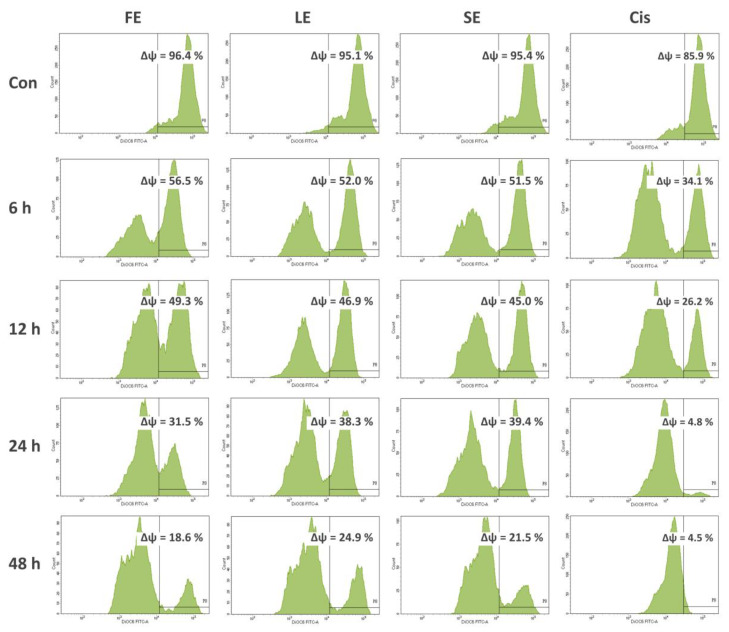
The changes of the mitochondrial membrane potential (ΔΨ) level in Caco-2 cells treated with broccoli extracts (0.20 mg/mL) and cisplatin (0.02 mg/mL) for various time points and analyzed using flow cytometry after staining with DiOC_6_ (3,3′-dihexyloxacarbocyanine iodide).

To determine whether extracts induced cell death by cell cycle arrest and/or apoptosis, Caco-2 cells were treated with extracts (0.20 mg/mL) and subjected to flow cytometry to analyze cell cycle distribution and mitochondrial membrane potential. The results are presented in Figure 1 and Figure 2.

Cell cycle analysis was undertaken to classify the cell population into four distinctive phases, comprising the subG1 phase, G0/G1 phase, S phase, and G2/M phase. The subG1 population is an indication of apoptotic cell death, and the two checkpoints are G1/S and G2/M transitions [19]. As shown in Figure 1, Caco-2 cells showed a significant increase of population in the subG1 phase from around 4% in the case of control to approximately 15% and 31% when exposed to broccoli extracts for 24 and 48 h, respectively. Moreover, the noticeable decreases of cell percentage in the G0/G1 phase after 24 h, and in the G2/M phase after 48 h of treatment, with a corresponding extension in the subG1 phase, confirmed the cycle arrest result. As a positive control, cisplatin-treated cells had the highest subG1 population, as well as obvious changes in other phases.

The apoptotic process is linked to the loss of mitochondrial membrane potential (MMP) by the activation of caspase-9 [20]. The effect of extracts on the level of MMP was examined to explore the mechanism of extract-induced apoptosis in Caco-2 cells. As shown in Figure 2, at 48 h of incubation, FE, LE, and SE significantly reduced the MMP level to 18.6%, 24.9%, and 21.5%, correspondingly, compared with about 95% of the untreated cells. The decrease appeared to exhibit a time-dependent manner. Besides, the serious loss of MMP level (from 85.9% to 4.5%) was found in cells exposed to cisplatin (0.02 mg/mL).

### 2.3. Antibacterial Activity

The antibacterial potential of broccoli extracts was investigated using the agar well diffusion and the broth microdilution techniques to observe inhibition zone diameters (IZDs) of bacterial growth around wells, and minimum inhibition concentrations (MIC) towards bacterial growth, respectively. The foodborne pathogens, including both Gram-negative bacteria (*Staphylococcus aureus* and *Bacillus subtilis*) and Gram-positive bacteria (*Escherichia coli* and *Salmonella typhimurium*), were selected for the study. Ampicillin and amoxicillin (0.1 mg/mL) were used as positive controls. For crude extracts of plants, IZDs were evaluated as the levels of inactive (<12 mm), moderately active (12–15 mm), active (15–21 mm), and highly active (>18 mm), whereas MICs were considered as the values of highly active (<0.1 mg/mL), active (0.1–0.5 mg/mL), moderately active (0.5–1.0 mg/mL), weak activity (1.0–8.0 mg/mL), and inactive (>8.0 mg/mL) [21,22].

The antibacterial properties of samples were presented by distinct means in Table 5 and Table 6. As shown in Table 5, FE and LE indicated stronger detrimental effects on foodborne bacteria than those of SE by obvious IZDs. All samples were highly active against *B. subtilis* and *S. typhimurium*, while the inhibitory of *S. aureus* and *E. coli* growth was at a level of moderately active. On the other hand, ampicillin and amoxicillin obtained very high values towards all bacterial strains. As shown in Table 6, LE effectively inhibited the growth of tested bacteria with the lowest MIC values in the range of 0.78–1.56 mg/mL. *B. subtilis* were found to be the most sensitive organisms to the effect of all broccoli extracts, in comparison with other strains tested.

## 3. Discussion

Every year, the agri-food industry generates a massive amount of food waste around the world. It leads to environmental and food security issues owing to the impacts related to the production and treatment [2]. The valorization of agriculture and food byproducts is an objective of the European Union to support sustainable development [8]. Previously, these agri-food residues were applied for animal feeds, compost, or essential oil extractions [1,3]. Presently, there is an increasing interest in the research associated with providing bioactive components of food byproducts as new ingredients for functional product formulations. Several studies investigated the chemical composition and biological activities of tomato leaves, orange peels, hazelnut shells, mango peels, or pomegranate marcs [4,5,6,7,23,24]. For example, orange peels revealed an anti-inflammatory bowel potential after 15 days of application in mice treated with dextran sodium sulfate [4]. The isolated proanthocyanidins from grape seeds possessed significant antioxidant and anti-inflammatory properties [6]. The extracts of pomegranate internal membranes by green technologies were used to enrich a cosmetic hydrophilic gel, as a model cosmetic [7]. Broad bean pods were demonstrated as a rich source of bioactive ingredients with antimicrobial, enzymes inhibitory, and anti-diabetic properties [25].

For functional product formulations, *Brassica* vegetables and their byproducts could be a valuable source of bioactive compounds and health benefits [9,18,26]. Numerous publications, counting our previous study, analyzed the bioactive composition and the potentially beneficial bioactivities of broccoli florets [9,12,27,28,29]. So far, the functional properties of broccoli byproducts have not been well documented. Some studies revealed the nutritional value, antioxidant, and antiproliferative activities of broccoli leaves and seeds [16,17,18]. Particularly, a report indicated that broccoli roots contained high levels of glucosinolates and related hydrolysis products, as well as provided information of the glucosinolate metabolome and transcriptome for distinct tissues of broccoli [30]. In the present study, LE and SE showed substantial polyphenolic concentrations, along with considerable antioxidant and cytotoxic activities on various in vitro models (Table 1, Table 2, Table 3 and Table 4). Additionally, corresponding with potential cytotoxicity towards cancer cells, our study confirmed the selective activity of broccoli extracts on the normal liver cells (FL83B). Nonetheless, this test was not previously detailed for all broccoli tissues.

Tumors are caused by the disorder of cell proliferation and the obstruction of cell apoptosis. Searching for bioactive compounds, which can induce cell apoptosis, would be an important strategy for potential chemotherapeutic agents [20]. Cancers are the consequence of cell-cycle dysregulation. Targeting the checkpoint signaling pathway, which generally leads to an arrest at the G1/S or G2/M phases, would be another effective therapeutic strategy [19]. Loss of MMP is closely linked to the activation of caspase-9, which plays a critical role in the initiation and maintenance of apoptosis [20]. Therefore, in this study, to evaluate if the cytotoxicity of broccoli extracts was the result of the induction of apoptosis or cell cycle arrest, Caco-2 cells were exposed to FE, LE, and SE and cell cycle distribution and MMP were analyzed using flow cytometry. The results showed significant increases in cell percentage with subG1 DNA content, obvious cell cycle arrests, as well as notable decreases of MMP (Figure 1 and Figure 2). To date, a few authors, along with our earlier publication [28], reported the mechanism of cancer cell death by the effects of broccoli, but focused on florets and sprouts only. A study presented the high efficacy of broccoli florets and sprouts on viability inhibitory and proapoptotic induction in human cancer cell lines [31]. Another study revealed the apoptotic role of isothiocyanate from broccoli florets in experimental lung carcinogenesis in Swiss albino mice [32].

Many reports demonstrated that the antimicrobial activity of plant extracts was attributed to the presence of bioactive compounds such as phenols, glucosinolates, or organic acids. Plant phenolics and extracts rich in such substances could inhibit the growth of various bacterial pathogens [33]. Over the last years, the detrimental effect on microorganisms was extensively studied on broccoli florets [34,35,36,37]. For instance, the antimicrobial activity of broccoli florets was evaluated using flow cytometry to identify dead cells probably owing to cell disruption and effusion of internal contents [36]. The capacity of florets against pathogenic bacteria, yeast, and fungi was also proven by the antimicrobial peptides [37]. However, there is no study available about the antimicrobial potential of broccoli leaves and seeds. This is the first study to present the antibacterial potency of LE and SE towards foodborne pathogens by the IZDs and MICs (Table 5 and Table 6). Briefly, together with our previous findings on broccoli sprouts [28], the present study indicated that both edible and non-edible parts of broccoli were effective for different biological activities.

## 4. Materials and Methods

### 4.1. Plant Materials and Preparation of Crude Extracts

Broccoli (*Brassica oleracea* L. var. *italica*) samples, Green King variety, were obtained from Known-You Seed Co. Ltd., Kaohsiung, Taiwan (22°39′39.8″ N 120°25′32.0″ E) and its experimental station, Tainan, Taiwan (23°04′46.5″ N 120°17′43.4″ E), in four periods, including November 2017, December 2017, March 2018, and November 2018. These are the end of the harvesting stages of broccoli, after being cultivated in conventional cool seasons in Taiwan. The samples were divided into three groups, comprising florets (a main edible part), leaves, and seeds (non-edible parts). Seeds in the original intact package were purchased directly from the company. Florets were harvested in 60–90 days after transplanting. Broccoli heads, which reached 4–8 inches in diameter with the dense and firm flower clusters, were collected. Thereafter, the side shoots stopped growing; this is a sign the plant is no longer producing, leaves were picked as unused byproduct. Mature leaves without signs of mechanical damage were selected. Each sample group was obtained from various plants for each biological replication.

Samples were dried in a TR 120 drying oven (Nabertherm GmbH, Lilienthal, Germany) at 40 °C, grounded into fine powders, and stored at −20 °C until extraction. Firstly, 10 g of sample powder was transferred into a flask containing separately 100 mL of each solvent (70% ethanol, 70% ethanol, or hot water) to reach the concentration of 0.1 g dry weight (DW)/mL. Ethanol and methanol extractions were performed by a SR-2 DW shaker (Taitec, Koshigaya-shi, Saitama-ken, Japan) for 24 h at 30 °C. Hot water extraction was undertaken by adding samples into boiling distilled water for 15 min. Extracts were centrifuged at 10,000 rpm for 10 min and supernatants were filtered through Whatman No.1 filter paper (Merck KGaA, Darmstadt, Germany). The filtrates were collected and stored at 4 °C for further polyphenolic content and antioxidant determinations [38,39]. Subsequently, the filtrates were concentrated to dryness using a RV8 rotary evaporator (IKA Works Guangzhou, Guangzhou, China) and an Alpha 1-2 LDplus freeze dryer (Martin Christ GmbH, Osterode, Germany). The lyophilized extracts were stored at 4 °C for further anticarcinogenic and antibacterial experiments.

### 4.2. Antioxidant Properties

#### 4.2.1. Antioxidant Activity Assays

For antioxidant properties, DPPH (2,2-diphenyl-1-picrylhydrazyl) radical scavenging, ABTS (2,2’-azino-bis-3-ethylbenzothiazoline-6-sulphonic acid) radical cation decolorization, and reducing power assays were utilized. Ascorbic acid solution (0.5 mg/mL) was employed as a positive standard.

The DPPH assay was performed as reported earlier [40]. Then, 1 mL of extracts (0.1 g DW/mL) was mixed with 4 mL of DPPH methanolic solution (0.1 mM), and incubated for 60 min to allow for complete reaction. The absorbance of mixtures was read at 517 nm, and results were calculated as the absorbance inhibition (%) of a blank composed of DPPH and methanol as extract solvent.

The ABTS radical cation was produced by mixing ABTS stock solution (7 mM) with potassium persulfate (2.45 mM) and kept at room temperature in the dark for 16 h before use. Then, 1 mL of diluted ABTS solution (0.70 absorbance at 734 nm) was mixed with 10 µL of extracts (0.1 g DW/mL). The absorbance of the mixtures was read at 415 nm. The antioxidant capacity was calculated based on the calibration curve and expressed as Trolox equivalents (µmol TE/g DW) [41].

The potency of extracts to reduce iron (III) was tested, as reported earlier [40]. Then, 1 mL of extracts (0.1 g DW/mL) reacted in 0.5 mL of 0.2 M phosphate-buffered saline (PBS) and 0.5 mL of 1% potassium ferricyanide (Fe^3+^). The reaction was stopped by adding 0.5 mL of 10% trichloroacetic acid. After centrifugation, 0.25 mL of 0.1% ferric chloride was added to 1 mL of supernatants to form ferric ferrous complex with an absorption maximum at 700 nm. The absorbance indicated reducing power.

#### 4.2.2. Determination of Total Phenolic, Flavonoid, and Vitamin C Contents

The total phenolic content (TPC) was determined by the Folin–Ciocaltecu method, as previously described [28]. The absorbance of the reaction mixture was measured at 765 nm using a SPECTROstar microplate reader (BMG labtech, Ortenberg, Germany). Gallic acid standards were applied to generate a calibration curve. TPC was expressed as gallic acid equivalent (mg GAE/g DW).

The total flavonoid content (TFC) was determined with aluminum chloride (AlCl_3_), as previously described [28]. The absorbance of the reaction mixture was measured at 490 nm. TFC was expressed as catechin equivalent (mg CE/g DW).

The vitamin C content (VCC) was determined using the Folin–Ciocalteu reagent, as previously described [28]. The absorbance of the reaction mixture was measured at 760 nm. VCC was expressed as ascorbic acid equivalent (mg AA/g DW).

#### 4.2.3. High-Performance Liquid Chromatography (HPLC) Analysis of Phenolic Compounds

The phenolic composition of ethanolic extracts was analyzed using an HPLC system (Hitachi Chromaster, Tokyo, Japan) according to the method previously described [28]. Sample compounds were separated in a NUCLEODUR^®^ C_18_ HTec column (250 × 4.6 mm, the particle size of 5 µm, Macherey-Nagel, Düren, Germany) at 25 °C by a flow rate of 1 mL/min. The mobile phase consisted of a mixture of 0.1% trifluoroacetic acid solution (A) and 100% methanol (B) with the following gradient: from 0–3 min, 10% B; from 3–20 min, 30% B; from 20–30 min, 40% B; from 30–50 min, 60% B; and from 50–60 min, 20% B. The eluted peaks were detected at 280, 320, and 360 nm. Phenolic compounds were identified and quantified by comparing the chromatographic behavior and retention times in specific UV spectra with external standards and reported data.

### 4.3. Anticancer Properties

#### 4.3.1. Cell lines and Culture Conditions

The selected cell lines for the cytotoxicity test were procured from the Food Industry Research and Development Institute (Hsinchu, Taiwan). Hepatocellular carcinoma (HepG2) and colorectal adenocarcinoma (Caco-2) cells were cultivated in Dulbecco’s modified Eagle’s medium, while lung carcinoma (A549) and normal liver (FL83B) cells were maintained in F12K medium. Cells were grown at standard conditions (37 °C, 5% CO_2_, 95% humidity) in the media supplemented with 10% fetal bovine serum, penicillin (100 U/mL), streptomycin (100 μg/mL), and sodium bicarbonate (1.5 g/L).

#### 4.3.2. Cytotoxic Assay

The cytotoxic activity was performed as described earlier [42]. Cells were seeded at 1 × 10^4^ cells per well in 96-well plates. After 24 h of incubation, cell media was removed and attached cells were stimulated with four concentrations of each extract (0.063–0.500 mg/mL) for 24 and 48 h. Cisplatin (0.006–0.050 mg/mL), a traditional chemotherapeutic agent, was treated similarly, as a positive control. Then, 20 μL of MTT (3-(4,5-dimethylthiazol-2-yl)-2,5-diphenyltetrazolium bromide) solution (5 mg/mL) was transferred to each well. After 2 h, supernatants were replaced with 100 µL of DMSO (dimethyl sulfoxide), and optical densities were measured at 570 nm. The results were expressed as a percentage of viable cells with respect to untreated cells, which was defined as 100%. The 50% inhibitory concentration (IC_50_) was calculated using the Graphpad Prism 5 software (GraphPad Software, San Diego, CA, USA).

#### 4.3.3. Cell Cycle Analysis

For flow cytometry analysis of DNA content, a total of 1 × 10^6^ Caco-2 cells were seeded in a 25 cm^2^ culture flask. After an overnight incubation, cells were exposed to extracts (0.200 mg/mL) and cisplatin (0.020 mg/mL) for 24 and 48 h. Cells were collected, washed with PBS, and fixed in cold 70% ethanol overnight at −20 °C. Cells were then washed twice in PBS at a centrifugation speed of 1500 rpm, resuspended in 1 mL of propidium iodide (PI) solution containing PI (0.050 mg/mL), 0.5% Triton X-100, and RNAse A (0.050 mg/mL), and incubated in the dark for 30 min at 37 °C [43]. The fluorescence intensity was analyzed by the FACS Calibur flow cytometer (BD Biosciences, San Jose, CA, USA). The results were expressed in a histogram as a total percentage of cells from four different cell cycle phases using the BD FACSDiva 8.0 software (BD Biosciences, San Jose, CA, USA).

#### 4.3.4. Assessment of Mitochondrial Membrane Potential (MMP)

DiOC_6_ (3,3′-dihexyloxacarbocyanine iodide), a cell-permeable fluorescent lipophilic dye, was used to assess MMP as reported [20]. After treatment with extracts (0.200 mg/mL) and cisplatin (0.002 mg/mL) for 6, 12, 24, and 48 h, Caco-2 cells were collected, washed with PBS, and stained with 1 mL of DiOC_6_ solution (0.5 µg/mL) in the dark for 30 min at 37 °C. The fluorescence intensity was analyzed by the FACS Calibur flow cytometer. The results were expressed in a histogram as a percentage of positive cells for the given dye, which represented MMP (ΔΨ), using the BD FACSDiva 8.0 software.

### 4.4. Antibacterial Properties

#### 4.4.1. Bacterial Strains and Culture Conditions

Two strains of gram-negative bacteria (*Staphylococcus aureus* CCRC 11863 and *Bacillus subtilis* CCRC 14199) and two strains of gram-positive bacteria (*Salmonella typhimurium* CCRC 12497 and *Escherichia coli* CCRC 11634) were used in this study. All these foodborne pathogens were obtained from the Culture Collection and Research Center (CCRC), Hsinchu, Taiwan. To obtain subcultures for further sample treatment, *S. aureus* and *E. coli* were cultured in the Tryptic soy broth, whereas *B. subtilis* and *S*. *typhimurium* were cultivated in the nutrient broth at 37 °C for 18 h.

#### 4.4.2. Agar Diffusion Method

The agar well diffusion method was firstly employed to evaluate antibacterial activity [44]. Agar plates were inoculated by spreading a 1% bacterial culture suspension (1 × 10^6^ CFU/mL) over the entire agar surface. On the agar plate surface, the wells with a diameter of 9 mm were then punched by a sterile cork borer, and 100 µL of extract solution (diluting extracts with 20% DMSO to reach a concentration of 50 mg/mL) was introduced into each well. Standard antibiotics (ampicillin and amoxicillin, 0.1 mg/mL) and negative control (20% DMSO) were included. Plates were incubated under suitable conditions depending upon the tested bacteria at 37 °C for 16 h. The results were expressed as the diameter (mm) of inhibition zones using a caliper.

#### 4.4.3. Broth Microdilution Method

The minimum inhibitory concentration (MIC) was determined by broth microdilution method [45]. Multiple two-fold dilutions of extracts (0.1–50 mg/mL) in the nutrient broth were performed in 96-well plates. Ampicillin and amoxicillin (0.1 mg/mL) were included as positive controls. A log-phase culture of the bacteria was diluted and inoculated into plates to a final concentration of 1 × 10^5^ CFU/mL. The plates were incubated at 37 °C for 16 h. Then, 20 μL of MTT (5 mg/mL) was transferred to each well as a colorimetric indicator of bacterial growth for 2 h. The MIC (mg/mL) was defined as the lowest concentration of extracts at which the tested bacteria showed no visible growth.

### 4.5. Statistical Analysis

All experiments were carried out in three replicates (*n* = 3). The results are presented as means ± standard deviation (SD). Statistical analysis was performed using SPSS 22.0 software (SPSS Inc., Chicago, IL, USA). The data were analyzed using one-way analysis of variance (ANOVA) with Tukey’s post hoc test for comparing mean significant differences between samples (*p* ≤ 0.05).

## 5. Conclusions

In the present study, both the edible and non-edible parts of broccoli were examined and compared in terms of antioxidant, cytotoxic, apoptotic, and antibacterial properties. LE showed the highest polyphenolic contents, correlated to its antioxidant activity. SE exhibited stronger cytotoxicity towards different cancer cell lines in comparison with those of FE and LE, without a toxic impact on normal cells. Importantly, LE and SE were presented for the first time as the mechanism of cell death induced by apoptosis and cell cycle arrest, as well as the antibacterial capacity against the tested foodborne pathogens. Therefore, rather than only utilizing florets, broccoli leaves and seeds were suggested as a source of bioactive ingredients for functional food and nutraceutical industries.

## Figures and Tables

**Table 1:** Antioxidant activity of edible and non-edible parts of broccoli.

Broccoli Parts	DPPH Scavenging Activity (Inhibition %)	Reducing Power Absorbance (700 nm)	ABTS Scavenging Activity (µmol TE/g DW)
FE			
70% Methanol	79.95 ± 2.63 ^bA^	1.04 ± 0.05 ^bB^	67.81 ± 0.17 ^aA^
70% Ethanol	79.37 ± 2.97 ^cA^	1.21 ± 0.03 ^cA^	67.92 ± 0.10 ^aA^
Hot water	58.21 ± 4.91 ^bB^	0.55 ± 0.07 ^cC^	43.06 ± 1.80 ^bB^
LE			
70% Methanol	81.64 ± 1.90 ^bA^	1.70 ± 0.02 ^aB^	68.23 ± 0.30 ^aA^
70% Ethanol	85.41 ± 1.32 ^bA^	1.79 ± 0.04 ^aA^	68.05 ± 0.25 ^aA^
Hot water	61.06 ± 9.15 ^bB^	0.84 ± 0.04 ^bC^	62.46 ± 0.87 ^aB^
SE			
70% Methanol	90.86 ± 0.65 ^aA^	1.65 ± 0.13 ^aA^	66.90 ± 0.27 ^bA^
70% Ethanol	92.13 ± 0.45 ^aA^	1.67 ± 0.06 ^bA^	68.51 ± 0.35 ^aA^
Hot water	79.53 ± 6.22 ^aB^	1.27 ± 0.05 ^aB^	64.77 ± 1.04 ^aB^
Ascorbic acid *	96.31 ± 1.08	2.59 ± 0.04	66.86 ± 0.21

DPPH, 2,2-diphenyl-1-picrylhydrazyl; ABTS, 2,2’-azino-bis-3-ethylbenzothiazoline-6-sulphonic; TE, Trolox equivalent; DW, dried weight; FE, floret extract; LE, leaf extract; SE, seed extract. * Positive control included (0.5 mg/mL). Data are expressed as mean values ± SD of triplicate experiments (*n* = 3). The ^a, b, c^ lowercase letters indicate significant differences between broccoli parts, and the ^A, B, C^ uppercase letters indicate significant differences between solvents at *p* ≤ 0.05.

**Table 2:** Antioxidant properties of edible and non-edible parts of broccoli.

Broccoli Parts	Total Phenolic Content (mg GAE/g DW)	Total Flavonoid Content (mg CE/g DW)	Vitamin C Content (mg AA/g DW)
FE			
70% Methanol	20.78 ± 1.09 ^bA^	5.32 ± 0.08 ^bB^	2.54 ± 0.35 ^aA^
70% Ethanol	19.50 ± 0.79 ^bA^	6.33 ± 0.21 ^bA^	2.50 ± 0.32 ^aA^
Hot water	15.35 ± 0.58 ^bB^	2.84 ± 0.13 ^cC^	1.66 ± 0.10 ^bB^
LE			
70% Methanol	28.50 ± 0.38 ^aA^	8.71 ± 0.16 ^aB^	2.92 ± 0.28 ^aA^
70% Ethanol	25.77 ± 0.37 ^a^^B^	9.93 ± 0.43 ^aA^	2.31 ± 0.10 ^aB^
Hot water	24.79 ± 0.32 ^aC^	7.84 ± 0.21 ^aC^	2.74 ± 0.12 ^aAB^
SE			
70% Methanol	16.55 ± 1.01 ^cA^	3.74 ± 0.10 ^cA^	2.69 ± 0.28 ^aA^
70% Ethanol	15.96 ± 0.87 ^cA^	3.51 ± 0.11 ^cA^	2.25 ± 0.39 ^aA^
Hot water	12.58 ± 0.54 ^cB^	3.59 ± 0.15 ^bA^	1.98 ± 0.33 ^bA^

GAE, gallic acid equivalent; CE, catechin acid equivalent; AA, ascorbic acid equivalent; the ^a, b, c^ lowercase letters indicate significant differences between broccoli parts, and the ^A, B, C^ uppercase letters indicate significant differences between solvents (*p* ≤ 0.05, *n* = 3).

**Table 3:** Levels of phenolic compounds in edible and non-edible parts of broccoli.

Phenolic Compounds (mg/g Extract)	FE	LE	SE
Gallic acid	0.526 ± 0.048 ^a^	0.150 ± 0.002 ^b^	0.165 ± 0.013 ^b^
Esculetin	4.573 ± 0.184 ^c^	6.488 ± 0.309 ^b^	10.179 ± 0.251 ^a^
Caffeic acid	0.795 ± 0.019 ^a^	ND	0.121 ± 0.008 ^b^
Ferulic acid	1.321 ± 0.087 ^a^	0.239 ± 0.003 ^c^	0.580 ± 0.016 ^b^
Myricetin	0.327 ± 0.004 ^b^	2.768 ± 0.129 ^a^	0.309 ± 0.014 ^b^
Quercerin	0.575 ± 0.003 ^b^	0.972 ± 0.017 ^a^	0.563 ± 0.016 ^b^

ND, not detected; the ^a, b, c^ lowercase letters indicate significant differences between broccoli parts (*p* ≤ 0.05, *n* = 3).

**Table 4:** Cytotoxic activity of edible and non-edible parts of broccoli.

Broccoli Parts	IC_50_ (mg/mL) *			
	HepG2	A549	Caco-2	FL83B
FE				
24 h	0.443 ± 0.048 ^aA^	0.318 ± 0.075 ^aA^	0.417 ± 0.039 ^aA^	>0.500
48 h	0.306 ± 0.052 ^aB^	0.184 ± 0.022 ^abA^	0.295 ± 0.032 ^bB^	>0.500
LE				
24 h	0.478 ± 0.026 ^aC^	0.257 ± 0.038 ^aA^	0.391 ± 0.015 ^aB^	>0.500
48 h	0.267 ± 0.090 ^aA^	0.191 ± 0.025 ^bA^	0.254 ± 0.013 ^abA^	>0.500
SE				
24 h	>0.500	0.271 ± 0.032 ^aA^	0.420 ± 0.035 ^aB^	>0.500
48 h	0.238 ± 0.031 ^aB^	0.134 ± 0.017 ^aA^	0.209 ± 0.016 ^aB^	>0.500
Cisplatin				
24 h	0.019 ± 0.001	0.009 ± 0.001	0.023 ± 0.002	>0.050
48 h	0.015 ± 0.003	<0.006	0.007 ± 0.003	0.027 ± 0.005

* IC_50_, a concentration in two-fold serial dilutions of extracts (0.063–0.500 mg/mL), which reduced cell growth by 50% after 24 and 48 h of treatment. HepG2, hepatocellular carcinoma cells; A549, lung carcinoma cells; Caco-2, colorectal adenocarcinoma cells; FL83B, normal liver cells. Cisplatin was referentially used as a positive control (0.006–0.050 mg/mL). The ^a, b^ lowercase letters indicate significant differences between broccoli parts at the same incubation time, and the ^A, B, C^ uppercase letters indicate significant differences between cell lines (*p* ≤ 0.05, *n* = 3).

**Table 5:** Antibacterial activity of edible and non-edible parts of broccoli.

Bacterial Strains	Diameter of the Inhibition Zones (mm) *					
	FE	LE	SE	Amp	Amo	D20
Gram-positive						
*S. aureus*	15.24 ± 0.40 ^bC^	17.16 ± 0.50 ^aB^	14.46 ± 0.45 ^bC^	36.38 ± 0.76	33.76 ± 0.56	ND
*B. subtilis*	26.79 ± 0.81 ^aA^	24.04 ± 0.66 ^bA^	25.81 ± 0.48 ^aA^	38.10 ± 0.75	37.33 ± 1.39	ND
Gram-negative						
*E. coli*	16.13 ± 0.25 ^aC^	16.20 ± 0.43 ^aB^	14.85 ± 0.64 ^bC^	39.04 ± 0.87	36.00 ± 0.60	ND
*S. typhimurium*	24.88 ± 0.92 ^aB^	23.51 ± 0.93 ^abA^	22.71 ± 0.32 ^bB^	43.54 ± 0.19	40.84 ± 0.47	ND

* Diameter of the inhibition zones (included 9 mm of well diameter) of broccoli extracts (50 mg/mL), positive controls: Amp, ampicillin; Amo, amoxicillin (0.1 mg/mL); negative control: D20, 20% DMSO (dimethyl sulfoxide). *S. aureus*, *Staphylococcus aureus*; *B. subtilis*, *Bacillus subtilis*; *S. typhimurium*, *Salmonella typhimurium*; *E. coli*, *Escherichia coli*. ND, not detected. The ^a, b^ lowercase letters indicate significant differences between broccoli parts, and the ^A, B, C^ uppercase letters indicate significant differences between strains (*p* ≤ 0.05, *n* = 3).

**Table 6:** Minimum inhibitory concentration (MIC) of edible and non-edible parts of broccoli.

Bacterial Strains	MIC (mg/mL) *			Control		
	FE	LE	SE	Amp	Amo	NT
Gram-positive						
*S. aureus*	1.56	1.56	3.13	−	−	+
*B. subtilis*	0.78	0.78	0.39	−	−	+
Gram-negative						
*E. coli*	3.13	3.13	3.13	−	−	+
*S. typhimurium*	1.56	0.78	1.56	−	−	+

* The MIC values were recorded in multiple two-fold dilutions of extracts (0.1–50 mg/mL). Positive controls: Amp, ampicillin; Amo, amoxicillin (0.1 mg/mL); negative control: NT, not treated by antibiotics or extracts. −, no bacterial growth; +, bacterial growth.
